# Development Of the VAMPCT Score for Predicting Mortality in CKD Patients with COVID-19

**DOI:** 10.7150/ijms.111558

**Published:** 2025-05-31

**Authors:** Chaofan Li, Yue Niu, Xinyan Pan, Dinghua Chen, Fei Liu, Zhe Feng, Yong Wang, Xueying Cao, Jie Wu, Jiabao Liu, Xin Guan, Xuefeng Sun, Li Zhang, Guangyan Cai, Xiangmei Chen, Ping Li

**Affiliations:** 1 Department of Nephrology, First Medical Center of Chinese PLA General Hospital, State Key Laboratory of Kidney Diseases, National Clinical Research Center for Kidney Diseases, Beijing Key Laboratory of Medical Devices and Integrated Traditional Chinese and Western Drug Development for Severe Kidney Diseases, Beijing Key Laboratory of Digital Intelligent TCM for the Prevention and Treatment of Pan-vascular Diseases, Key Disciplines of National Administration of Traditional Chinese Medicine (zyyzdxk-2023310), Beijing 100853, China.; 2 Department of Endocrine, Hebei General Hospital, Hebei 050051, China.; 3 Department of Urology, Chinese Academy of Medical Sciences and Peking Union Medical College, Beijing, China.

**Keywords:** chronic kidney disease, COVID-19, cohort study, machine learning, mortality.

## Abstract

**Background:** Chronic kidney disease (CKD) patients with coronavirus disease 2019 (COVID-19) are at significant risk of death. However, clinical identification of high-risk individuals remains suboptimal despite the recognition of many pathophysiological and comorbidity-related risk factors. We aim to develop a clinically simple machine learning (ML)-based score to predict acute COVID-19 mortality among CKD patients.

**Methods:** CKD inpatients with COVID-19 were prospectively enrolled from December 2022 to January 2023 with a three-month follow-up. Feature selection from clinical and laboratory results was performed through least absolute shrinkage and selection operator and stepwise selection. Logistic regression, support vector machine (SVM), random forest, and extreme gradient boosting were applied for ML model development. A predictive score for mortality was constructed using logistic regression. We compared predictive ability between the proposed score and other published scores.

**Results:** 219 CKD patients were included and had a high mortality rate of 25.1%. The SVM model exhibited the best performance, with the validation area under the receiver operating characteristic curve (AUC) being 0.946 (95% CI 0.918, 0.974). The COVID-19 vaccination status, age, monocyte percentage, prothrombin activity, cardiac troponin T, and total bilirubin (“VAMPCT”) were the most relevant factors and utilized to develop the scoring system with an AUC of 0.960 (95% CI 0.935, 0.985).

**Conclusion:** ML models predicting three-month mortality had favorable performance for CKD patients with COVID-19. The VAMPCT mortality score provided a user-friendly approach.

## Introduction

According to the report from the World Health Organization, although coronavirus disease 2019 (COVID-19) no longer constitutes a public health emergency of international concern, there are still ongoing reports of new infections and deaths related to severe acute respiratory syndrome coronavirus 2 (SARS-CoV-2) variants around the world, which have also contributed to the overall COVID-19 burden with varying magnitudes 1-3. Official data indicate that 90% of COVID-19-related in-hospital fatalities in China involved individuals with pre-existing medical conditions 4. Chronic kidney disease (CKD) is acknowledged as a significant comorbidity that predisposes individuals to a heightened risk of contracting SARS-CoV-2 and experiencing adverse outcomes, including increased mortality rates 5, 6. The Omicron variants sustained dominance in the global and Chinese COVID-19 landscapes. The characteristics of the COVID-19 acute phase and its impact on the CKD population in China during the Omicron wave are not well understood. Meanwhile, studies indicate that the initial three months post-infection are the peak period for mortality 7. Considering deaths within a three-month period post-infection as COVID-19-associated provides a more precise measure of the disease's impact. Furthermore, CKD patients are at elevated risk for viral infections, with factors influencing poor outcomes from SARS-CoV-2 potentially applicable to other viral infections in this group 8.

The full automation of ML processes has streamlined the development of models that are not only simple and rapid but also easily replicable, ensuring consistency and reliability. These models have proven to be more efficient than traditional, manually crafted models, offering significant advantages in supporting clinical decision-making and the strategic deployment of healthcare resources.

Consequently, we aimed to construct and validate a predictive scoring system utilizing machine learning techniques designed to pinpoint high-risk CKD patients who may benefit from timely interventions of COVID-19, thereby enhancing their overall prognosis during the Omicron wave.

## Methods

### Participants and setting

**Figure 1:**
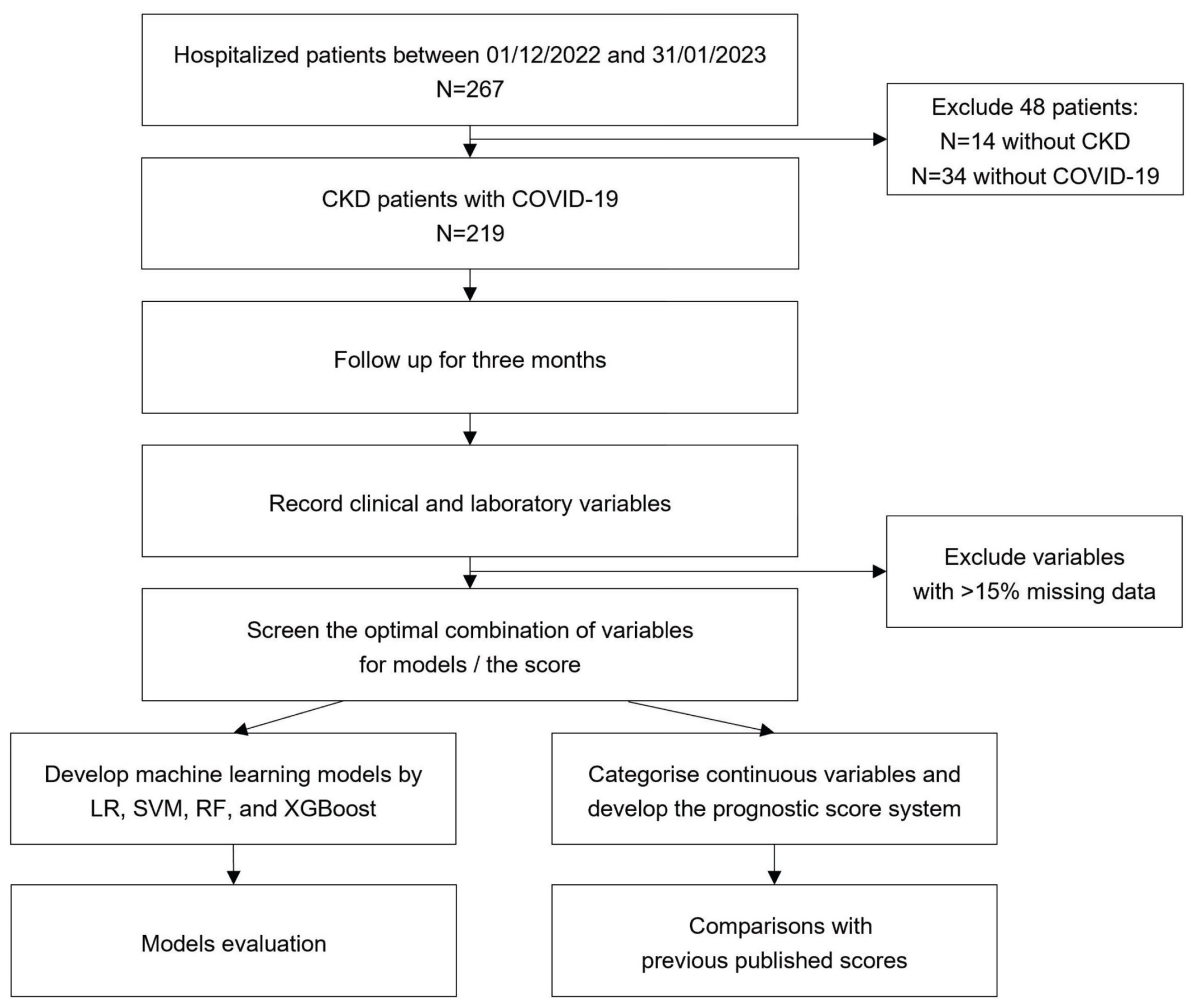
** Flow chart of the study.** CKD: chronic kidney disease; COVID-19: coronavirus disease 2019; LR: logistic regression; SVM: support vector machine; RF: random forest; XGBoost: extreme gradient boosting.

The prospective cohort study consecutively enrolled CKD inpatients with COVID-19 during the Omicron period from December 1, 2022 to January 31, 2023 at the Chinese People's Liberation Army General Hospital (PLAGH) (shown in Figure 1).

### Data collection and variable definition

Data extraction was performed from the electronic health records within the hospital information system at the PLAGH 9. The date of admission was designated as the index date for all enrolled patients. Comprehensive reviews of clinical charts, nursing notes, laboratory results, and radiological imaging were conducted.

Patients aged over 18 years were required to meet both diagnostic criteria for CKD (defined by the guideline of “Kidney Disease: Improving Global Outcomes” organization”) and COVID-19. Patients with extensive missing data or inability to complete follow-up were excluded. CKD is defined as abnormalities of kidney structure or function, present for a minimum of three months, with implications for health 10. The diagnostic criteria for COVID-19 involve the presence of clinical manifestations associated with SARS-CoV-2 infection and the fulfillment of at least one of the following etiological or serological test results: a positive SARS-CoV-2 nucleic acid test, a positive SARS-CoV-2 antigen test, successful isolation and culture of SARS-CoV-2, or SARS-CoV-2-specific IgG antibody levels in the convalescent phase being fourfold or higher than those in the acute phase, which adhered to the criteria outlined in the 10th edition of the Diagnosis and Treatment Protocol for COVID-19, as issued by the National Health Commission of China 1. In accordance with the guideline, patients acceped conservative or non-conservative treatment according to their disease severity. Conservative management included symptomatic support (e.g., hydration, oxygen therapy), while non-conservative interventions encompassed pharmacologic therapies such as glucocorticoids, Nirmatrelvir/Ritonavir, Azvudine, Baricitinib, or Tocilizumab.

The individual vaccination status was categorized into three groups: unvaccinated, partially vaccinated, and fully vaccinated. Full vaccination was defined as receiving at least one dose of the adenovirus vector vaccine, two doses of the inactivated vaccine, or three doses of the recombinant protein vaccine. CKD was identified according to the KDIGO guideline for CKD 10. Laboratory data included a complete blood count, coagulation profile, infection-related indicators, serum biochemical tests (including renal and liver function, creatine kinase, lactate dehydrogenase (LDH), and electrolytes), and cardiac biomarkers (such as troponin, brain natriuretic peptide, and myoglobin).

This retrospective cohort study analyzed the prognostic performance of the score across these subgroups to calculate odds ratios for 3-month mortality. Interaction terms were included to evaluate whether treatment modality modified the predictive utility of the score.

### Outcome

The clinical outcome was all-cause mortality confirmed by vital status at discharge, outpatient visits, or telephone follow-up during the three months after the admission. Patients were followed up and rightly censored on May 1, 2023.

### Data processing and variables selection

Variables with more than 15% missing values have not been considered. Multiple imputation was used to handle missing values on candidate variables, considering them missing at random (Table S1). Numeric variables were standardized based on the mean and variance. Least absolute shrinkage and selection operator (LASSO) regression and stepwise selection regression were performed for screening features to optimize the performance of machine learning models.

### Models and the score system development

The selected variables were fitted with ML algorithms including logistic regression (LR), support vector machine (SVM), random forest (RF), and extreme gradient boosting (XGBoost). To create the pragmatic mortality score, six variables that contributed the most to the outcome were further filtered out. Continuous variables were converted to dichotomous variables whose cut-off values were chosen by component smoothed functions from generalized additive modeling. The coefficients of logistic regression were converted into prognostic indexes for developing a practical score system.

### Model evaluation

Discrimination was evaluated using the area under the curve (AUC) of the receiver operator characteristic (ROC). We also assessed the corresponding Youden indexes, sensitivity, specificity, positive predictive values, and negative predictive values. The calibration was evaluated by the Hosmer-Lemeshow (H-L) test and calibration plot. The model's performance was rated using accuracy, F1 score, kappa coefficient, and Brier score. Additionally, decision curve analysis (DCA) was carried out to determine the clinical utility and calculate the net benefits at different threshold probabilities. All results underwent leave-one-out cross-validation for internal validation. Sensitivity analyses were performed by using complete case data and multiple imputation with different random seeds for missing data. The prognostic performance of the predicted score across treatment subgroups was evaluated to calculate odds ratios for 3-month mortality.

### Comparison with previous scores

In this study, "International Severe Acute Respiratory and Emerging Infections Consortium Coronavirus Clinical Characterization Consortium" (4C) mortality score, "Confusion, Urea, Respiratory rate, Blood pressure, and age ≥ 65 years" (CURB65) score, “Hypertension, Neutrophil count, C-reactive protein, Lymphocyte count, Lactate dehydrogenase” (HNC-LL) score, "quick Sequential Organ Failure Assessment" (qSOFA), and "Modified Early Warning Score" (MEWS) were calculated for each patient 11-14. The mortality score generated from this dataset was compared with the above-mentioned ones.

### General statistical analysis

The mean and standard deviation were used to represent normally distributed data, and independent t-tests were used to compare them. The Mann-Whitney test was used to compare non-normally distributed data that were reported as median (25%-75% interquartile range). Categorical variables were expressed as counts and percentages and tested using the chi-square test. A two-sided P <0.05 was considered statistically significant.

### Statistical software

All analyses were conducted with R 4.2.0 via packages including caret version 6.0-93, mice version 3.15.0, randomForest version 4.7.1.1, e1071 version 1.7-13, xgboost version 1.7.3.1, glmnet version 4.1.6, pROC version 1.18.0, and ggplot2 version 3.4.1.

### Ethical approval

The study was carried out in accordance with the Helsinki Declaration. It was authorized by the Ethics Committee of the Chinese PLAGH (S2023-111-01). All patients provided written informed consent prior to participation.

## Results

### Patients' characteristics

In our study, encompassing 219 participants, the majority were male (69.4%) with an average age of 59 years, and nearly half (47.5%) were 60 years of age or older (Table 1).

The average body mass index (BMI) was 23.95 kg/m². A significant portion, 63.5%, suffered from advanced CKD stages (four or five). Prior to the infection, 32.4% were on maintenance dialysis, while 5.5% had undergone kidney transplantation without dialysis. Hypertension was the predominant comorbidity at 77.2%, with cardiovascular disease (CVD) and diabetes mellitus following at 47.5% and 37.9%, respectively.

Vaccination rates against SARS-CoV-2 were suboptimal, with only 39.7% vaccinated, of which 36.0% had completed the basic vaccination schedule. The finger oxygen saturation on air of 23.3% of patients was below 90%. The median length of follow-up was 93 days. 74.9% (n = 164) of patients survived, whereas 25.1% (n = 55) deceased. The death group was older than the survivor group (76 ± 13 years vs. 53 ± 18 years, P<0.001). They displayed lower BMI (22.21 ± 3.94 kg/m^2^ vs. 24.53 ± 4.10 kg/m^2^, P<0.001), a higher proportion of combined CVD (76.4% vs. 37.8%, P<0.001), and cerebrovascular disease (18.2% vs. 6.7%, P = 0.025). The unvaccinated rate in the deceased was significantly higher at 89.1% versus 50.3% in survivors (P<0.001). At admission, systolic blood pressure (SBP) (131 ± 25 mmHg vs. 142 ± 24 mmHg, P = 0.006) and diastolic blood pressure (72 ± 13 mmHg vs. 80 ± 16 mmHg, P = 0.002) were lower in the death group than those in the survivor group. The proportion of finger oxygen saturation on air <90% (49.1% vs. 14.6%, P<0.001) was significantly higher in the death group than that in the survivor group.

### Variables selection

Through subsequent cross-validation with ML algorithms, the variable combination with the best performance was selected for modeling. Eleven variables were retained: age, SBP, COVID-19 vaccination status (Vacc), CVD, red blood cell volume distribution width (RDW), hematocrit (HCT), percentage of monocytes (mono), prothrombin activity (PTA), LDH, total bilirubin (TBil), and cardiac troponin T (cTnT).

### Model development and evaluation

**Figure 2:**
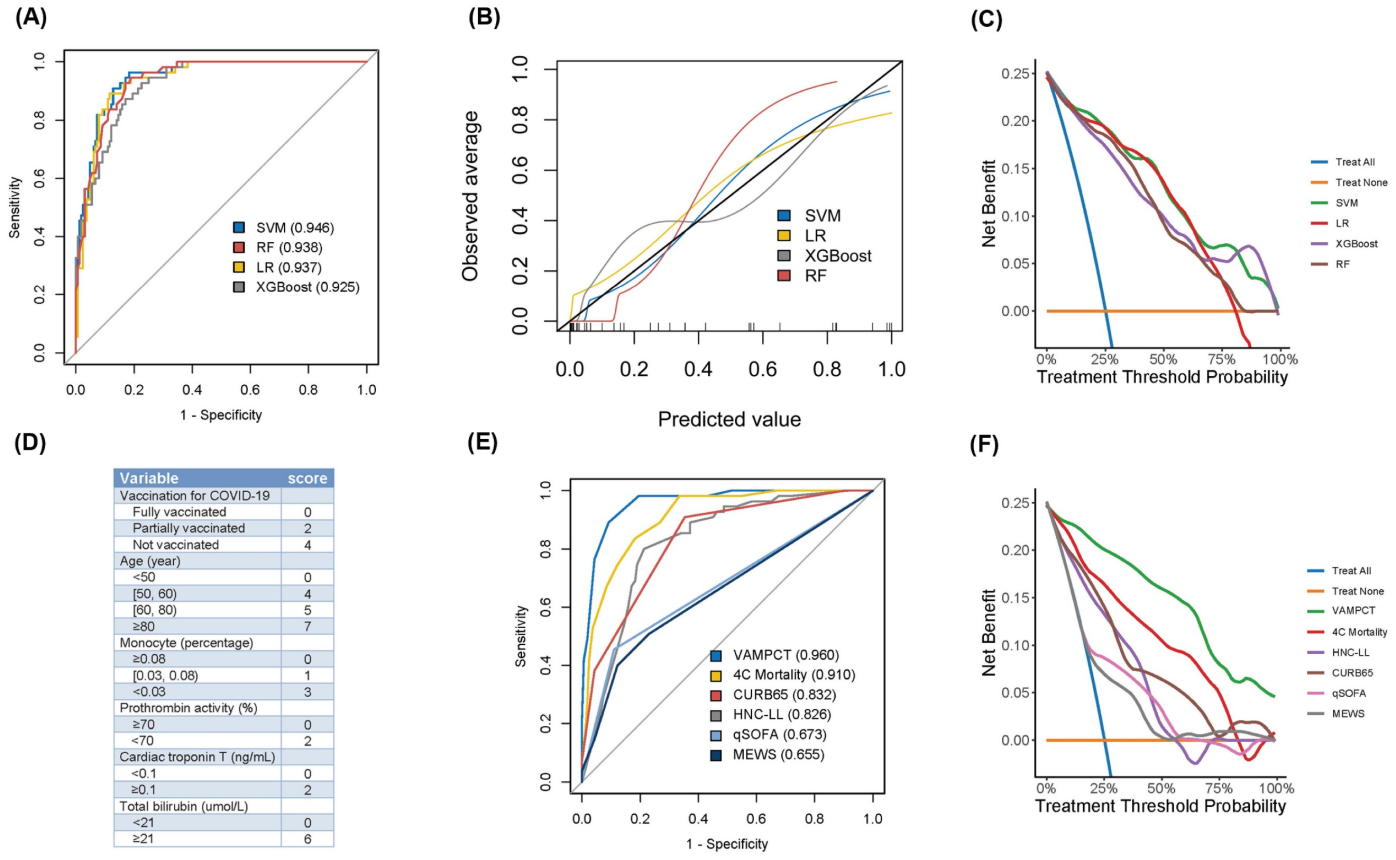
** The evaluation of predictive machine learning models and scores for three-month mortality in CKD patients with COVID-19. (A)** ROC analysis of four machine learning models.** (B)** The calibration plot of four machine learning models.** (C)** DCA of four machine learning models.** (D)** The predictive VAMPCT score.** (E)** ROC analysis of six predictive scores.** (F)** DCA analysis of six predictive scores. ROC: receiver operating characteristic; DCA: decision curve analysis; SVM: support vector machine; LR: logistic regression; XGBoost: extreme gradient boosting; RF: random forest; COVID-19: coronavirus disease 2019; 4C: Coronavirus Clinical Characterisation Consortium; HNC-LL: hypertension: neutrophil count: C-reactive protein: lymphocyte count: and lactate dehydrogenase; CURB65: confusion: urea: respiratory rate: blood pressure: and age ≥ 65 years; qSOFA: quick sequential organ failure assessment; MEWS: modified early warning score. The values in parentheses were the area under the curve.

Four ML models, including SVM, LR, RF, and XGBoost, were finally developed and tested with leave-one-out cross-validation. As the ROC curves shown in Figure 2A, the SVM model yielded better discrimination to predict the mortality of patients than other ML models (Table 2). The AUC (95% CI) and the Youden index of the SVM model were 0.946 (0.918, 0.974) and 0.781, respectively. Moreover, the Brier score of the SVM model was the lowest at 0.082 among the four models. For each ML model, calibration performance was further evaluated. The P values of H-L tests for both SVM and XGBoost models were all >0.05. Graphically, the calibration plot of the SVM model fitted well with the diagonal reference line (shown in Figure 2B). Generally, the SVM model had better calibration performance than the other models. As shown in Figure 2C, DCA was applied for assessing the clinical benefits, and the SVM model performed better than the others. It still revealed net benefits when approaching the 100% threshold probability. Based on the above evaluations from three aspects, the SVM model had the best predictive performance among the four ML models when predicting the mortality of CKD patients with COVID-19.

### The three-month mortality score

Given the need to use pragmatic scores at the bedside, the number of variables was reduced, and we identified six significant predictors of mortality as Vacc, age, mono, PTA, cTnT, and TBil (for short as “VAMPCT”). The continuous variables were transformed into factors with cut-off values (shown in Figure S1). Age was stratified into four categories: less than 50 years old, 50 to 60 years old, 60 to 80 years old, and 80 years old or older. The percentage of monocytes was divided into three tiers: not less than 0.08, 0.03 to 0.08, and less than 0.03. The PTA was bifurcated at the threshold of 70. Similarly, cTnT and TBil were stratified into two levels using the cut-offs of 0.1 and 21, respectively. Logistic regression was used to construct a risk score, and the regression coefficients were converted into a prognostic index by using appropriate scaling. As shown in Figure 2D, the total scores of VAMPCT ranged from 0 to 24. In the derivation cohort, the VAMPCT score showed a good discrimination of mortality within three months (AUC 0.960, 95% CI 0.935, 0.985), which was better than the existing scores (4C mortality score, CURB65 score, HNC-LL, qSOFA, and MEWS) (shown in Figure 2E-F and Table S2). DCA analysis showed that the VAMPCT score had better clinical utility across a wide range of thresholds. In general, the VAMPCT score outperformed the existing risk scores in predicting three-month mortality. According to the ROC analysis, two risk groups were defined with the optimal cut-off value determined (Table S3): low risk (0-10 score, mortality rate 3.87%) group and high risk (≥ 11 score, mortality rate 76.56%) group.

### Sensitivity analysis

In the development of ML models, analyses with complete-data instances and alternative imputed cases produced findings comparable to those from the primary imputed dataset (Table S4). In the development of predictive scores, the analysis of forest plots with complete-data cases, distinct imputed instances, and in-hospital outcomes revealed significant P values and coefficients that were similar to the primary analysis (Figure S2).

### Subgroup analysis

As shown in Figure S3, subgroup analysis based on treatment modality demonstrated that the VAMPCT score effectively predicted three-month mortality in CKD patients with COVID-19 across both subgroups. In the conservative treatment-only group, the OR was 3.04 (95% CI: 1.68-8.71, P = 0.006), while in the group receiving at least one non-conservative treatment, the OR was 2.73 (95% CI: 1.97-4.21, P < 0.001). However, no statistically significant interaction was observed between treatment modality and the predictive performance of the VAMPCT score (P = 0.804), suggesting that its prognostic utility remained consistent regardless of treatment strategy.

## Discussion

The relentless global spread and mutational evolution of SARS-CoV-2 have posed profound threats to both human health and the social economy. In China, the validated genome sequences of SARS-CoV-2 have all been Omicron variants since December 2022 15. Notably, infections with the Omicron variant have been associated with reduced hospitalization and mortality rates compared to earlier variants of concern 16. According to the latest epidemiological survey, there were 82 million adults with CKD in China 17. A recent meta-analysis of 12 studies revealed that the mortality rate among CKD patients with COVID-19 was alarmingly 5.81 times higher than among those without infection 18. Highlighting the urgency of early identification of CKD patients at risk of severe outcomes is essential. This study, through an analysis of acute phase infection characteristics and subsequent follow-up of CKD patients, aimed to pinpoint risk factors and formulate a predictive model for mortality of COVID-19 during the Omicron wave.

In our study, all-cause mortality among patients with CKD at three months after COVID-19 was 25.1%, which varies from different studies. According to a multicenter cohort study, the 12-week mortality rate of COVID-19 patients with CKD was 41.5% 19. In Turkish, the mortality of CKD patients at three months after the diagnosis of COVID-19 was 5.2% 20. Several explanations may elucidate these variances. Principally, our study's patient population was largely affected by the Omicron variant, which is characterized by a reduced severity and mortality profile relative to its predecessors 21. Additionally, racial disparities could play a pivotal role in post-COVID-19 mortality, attributed to a spectrum of factors including distinct comorbidities and divergent biochemical progressions 22, 23.

In our analysis, eleven predictors were meticulously selected and applied through machine learning algorithms, capturing a comprehensive profile of COVID-19's impact. These predictors encompassed indicators of cardiac injury (cTnT and LDH), coagulation dysfunction (PTA), erythrocyte abnormalities (RDW), and the involvement of the immune system, including COVID-19 vaccination status and monocyte percentage. These parameters are not only routinely measured but also corroborate established risk factors for COVID-19 mortality as identified in previous studies 24-26. Within our cohort, age emerged as the most significant predictor of mortality. A wealth of evidence supports the association between advanced age and adverse outcomes in COVID-19 patients with CKD 27, 28. The interplay of a milder inflammatory response with aging, slower viral clearance, and the diminished compensatory capacity of the remaining glomeruli likely underpins this association 29-31. Frailty, a prevalent geriatric syndrome, is strongly associated with aging and portends elevated mortality in CKD patients, particularly when compounded by COVID-19. Mechanistically, age-related senescence involves subcellular/cellular perturbations—inflammaging, mitochondrial dysfunction, cellular senescence, and dysregulated nutrient-sensing pathways—culminating in multisystem physiological decline and clinical frailty 32. In CKD patients, frailty and COVID-19 synergistically amplify proinflammatory cascades, further impairing antiviral immunity while exacerbating hyperinflammation-driven organ injury, thereby increasing severe disease and mortality risks 33. Frailty also compromises tolerance to SARS-CoV-2-targeted antivirals (e.g., nirmatrelvir/ritonavir), necessitating dose modifications or alternative regimens that may undermine therapeutic efficacy 34.

Incorporating cardiac biomarkers into the scoring system is critical, given the high prevalence of cardiovascular comorbidities (e.g., hypertension, diabetes, coronary artery disease) and compounded cardiorenal risks in CKD patients 35. Meanwhile, COVID-19 exacerbates these risks through direct myocardial injury (ACE2-mediated viral entry) and systemic hyperinflammation, increasing acute complications like myocarditis and thrombosis. Previous clinical studies have implied that COVID-19 leads to diverse cardiovascular complications 36. Biomarkers such as troponin refine prognostic accuracy by quantifying these interactions, enabling early intervention to mitigate mortality. Thus, cardiovascular-integrated scoring addresses the unique pathophysiology of CKD-COVID-19 overlap, improving both risk prediction and personalized management.

Vaccination has been heralded as a pivotal preventive measure in mitigating the severity and reducing fatalities from COVID-19 37. Our findings underscore vaccination status as the most potent protective factor, a consensus echoed by prior research. A multicenter study highlighted that the relative risk of death for vaccinated individuals 90 days post-COVID-19 was a fifth of that for their unvaccinated counterparts 38. Similarly, in the hemodialysis population, vaccination has been linked to attenuated disease severity and lower mortality rates attributable to COVID-19 39.

Advanced machine learning (ML) techniques have unlocked the potential to uncover subtle patterns within the intricate and high-dimensional landscape of clinical data. In terms of the AUC, our ML models demonstrated exceptional performance, a testament to the effectiveness of feature selection as well as the meticulous training and tuning processes employed. When considering calibration and clinical applicability, support vector machine (SVM) models emerged with a more advantageous overall performance, a finding that aligns with reports on COVID-19 patient outcomes 40, 41. A recent meta-analysis has pointed out that the algorithm used, the population studied, the study design, and the dataset source all exert influence on the pooled estimate of model performance 42. With clinical practicality in mind, we distilled six impactful indicators from those identified by ML to develop the "VAMPCT" scoring system. This scoring system offers predictive discrimination comparable to the SVM model, coupled with enhanced specificity, thereby facilitating its utility in clinical decision-making.

Despite the robust findings of our study, several limitations warrant acknowledgment. Firstly, the data were sourced from a single hospital, and the modest sample size may constrain the robustness of the machine learning model scoring and the generalizability of our results to other geographic regions or ethnic groups, where variations in healthcare practices, genetic predispositions, and COVID-19 strain prevalence could influence prognostic accuracy. Prospective validation in multiethnic, multinational cohorts is required to confirm its broader applicability. Secondly, our analysis relied on multiple imputation under the assumption of data missing at random, which may not accurately reflect the true distribution patterns; this assumption could introduce bias. Thirdly, our findings may be influenced by residual confounding from unmeasured factors (e.g., socioeconomic status, lifestyles, and behaviors) and imperfectly modeled nonlinear/interaction effects. While sensitivity analyses supported robustness, future prospective studies with granular phenotyping are needed to fully address these limitations. These limitations should be considered when interpreting the study outcomes and when planning subsequent research to address these gaps.

## Conclusion

In this study, we developed predictive models for three-month mortality in CKD patients with COVID-19, identifying the SVM model as the most effective. We also introduced the VAMPCT score to facilitate early prognostic evaluation during the acute phase of the disease. Against the backdrop of the Omicron variant's sustained dominance in the global and Chinese COVID-19 landscapes, our research offers initial observations regarding the mortality associated with Omicron infection in CKD patients. It contributes to paving the way for the advancement of more refined and prognostically relevant clinical tools.

## Supplementary Material

Supplementary figures and tables.

## Figures and Tables

**Table 1:** Clinical characteristics of CKD patients with COVID-19 according to the outcomes

Characteristic	Total (N=219)	Survivor (N=164)	Death (N=55)	P value
Age (year)	59 ± 19	53 ± 18	76 ± 13	< 0.001
Sex				0.571
Male	152 (69.4)	116 (70.7)	36 (65.5)	
Female	67 (30.6)	48 (29.3)	19 (34.5)	
Body mass index (kg/m^2^)	23.95 ± 4.18	24.53 ± 4.10	22.21 ± 3.94	< 0.001
CKD stages				0.005
CKD 1	20 (9.1)	20 (12.2)	0 (0.0)	
CKD 2	23 (10.5)	22 (13.4)	1 (1.8)	
CKD 3	37 (16.9)	26 (15.9)	11 (20.0)	
CKD 4	34 (15.5)	23 (14.0)	11 (20.0)	
CKD 5	105 (47.9)	73 (44.5)	32 (58.2)	
Diagnosis of CKD				<0.001
IgA nephropathy	23 (10.5)	23 (14.0)	0 (0.0)	
Diabetic nephropathy	18 (8.2)	15 (9.1)	3 (5.5)	
Membranous nephropathy	13 (5.9)	13 (7.9)	0 (0.0)	
Other CGN^a^	82 (37.4)	52 (31.7)	30 (54.5)	
Renal replacement^b^	83 (37.9)	61 (37.2)	22 (40.0)	
Comorbidities				
Hypertension	169 (77.2)	125 (76.2)	44 (80.0)	0.695
Cardiovascular disease	104 (47.5)	62 (37.8)	42 (76.4)	< 0.001
Diabetes mellitus	83 (37.9)	57 (34.8)	26 (47.3)	0.135
Cerebrovascular disease	21 (9.6)	11 (6.7)	10 (18.2)	0.025
Cancer	21 (9.6)	13 (7.9)	8 (14.5)	0.239
Vaccination for COVID-19^c^				< 0.001
Unvaccinated	129 (60.3)	80 (50.3)	49 (89.1)	
Partially vaccinated	8 (3.7)	6 (3.8)	2 (3.6)	
Fully vaccinated	77 (36.0)	73 (45.9)	4 (7.3)	
Admission vitals				
Body temperature (℃)	36.5 (36.3-36.8)	36.5 (36.3-36.7)	36.5 (36.4-36.8)	0.187
Heart rate (beats/min)	86 ± 15	85 ± 14	86 ± 18	0.669
Systolic blood pressure (mmHg)	139 ± 24	142 ± 24	131 ± 25	0.006
Diastolic blood pressure (mmHg)	78 ± 16	80 ± 16	72 ± 13	0.002
Finger oxygen saturation on air < 90%	51 (23.3)	24 (14.6)	27 (49.1)	< 0.001
Laboratory test				
Red blood cell (10^12^/L)	3.41 ± 0.94	3.42 ± 0.92	3.39 ± 0.99	0.807
Hemoglobin (g/dL)	10.42 ± 2.81	10.43 ± 2.83	10.40 ± 2.77	0.945
RDW (%)	13.99 ± 1.98	13.62 ± 1.65	15.13 ± 2.43	< 0.001
White blood cell (10^9^/L)	6.50 (4.81-9.39)	6.08 (4.58-8.32)	7.54 (6.08-11.80)	< 0.001
Neutrophil (percentage)	0.74 ± 0.14	0.70 ± 0.13	0.84 ± 0.12	< 0.001
Lymphocyte (percentage)	0.16 ± 0.11	0.19 ± 0.11	0.09 ± 0.07	< 0.001
Monocyte (percentage)	0.08 ± 0.04	0.09 ± 0.03	0.06 ± 0.04	< 0.001
Platelet (10^9^/L)	190.32 ± 85.19	201.49 ± 87.68	157.04 ± 67.68	0.001
Serum albumin (g/L)	31.42 ± 6.64	31.93 ± 7.17	29.91 ± 4.44	0.051
Blood urea (mmol/L)	17.45 (10.50-27.51)	15.73 (8.72-24.41)	25.15 (16.45-39.74)	< 0.001
Serum creatinine (μmol/L)	403.00 (158.00-761.13)	365.10 (130.2-796.5)	457.2 (219.6,723.0)	0.234
eGFR (mL/min/1.73 m^2^)	11.25 (5.60-42.20)	13.68 (5.69-48.34)	9.19 (5.36-21.33)	0.016
C-reactive protein (mg/dL)	1.53 (0.16-6.04)	0.39 (0.10-2.67)	8.35 (2.81-11.77)	< 0.001
Interleukin-6 (pg/mL)	13.93 (3.02-63.35)	6.04 (2.29-30.25)	103.95 (27.99-192.62)	< 0.001
Lactate dehydrogenase (U/L)	232.80 (189.70-336.50)	217.90 (172.48-272.10)	339.50 (251.30-438.25)	< 0.001
Prothrombin activity (%)	92.45 ± 22.30	98.66 ± 18.95	73.83 ± 21.32	< 0.001
APTT (s)	37.50 (34.18-43.60)	36.80 (33.80-41.85)	40.55 (36.95-46.90)	< 0.001
Plasma fibrinogen (g/L)	4.87 ± 1.70	4.72 ± 1.57	5.34 ± 2.00	0.019
D-dimer (μg/mL)	1.34 (0.58-2.54)	1.02 (0.44-2.02)	2.65 (1.59-5.49)	< 0.001
BNP (pg/mL)	5196.00 (573.15-21150.50)	3773.50 (359.40-16038.50)	10630.00 (2031.00-28398.50)	0.002
Myoglobin (ng/mL)	171.65 (70.28-338.00)	120.10 (58.90-221.00)	259.60 (168.45-639.45)	< 0.001
Cardiac troponin T (ng/mL)	0.06 (0.02-0.12)	0.04 (0.01-0.09)	0.11 (0.07-0.15)	< 0.001
Time from onset to admission (day)	14 (7-32)	18 (10-38)	7 (2-14)	< 0.001
Length of hospital stay (day)	15 (8-32)	16 (8-31)	13 (6-35)	0.331

Data are expressed as number (%), mean ± standard deviation, or median (interquartile range). CKD: chronic kidney disease; COVID-19: coronavirus disease 2019; CGN: chronic glomerulonephritis; RDW: red blood cell volume distribution width; eGFR: estimated glomerular filtration rate; APTT: activated partial thromboplastin time; BNP: brain natriuretic peptide. a: Other CGN: minimal change disease (9, 4.1%), anti-neutrophil cytoplasmic antibodies-associated glomerulonephritis (4, 1.8%), focal segmental glomerulosclerosis (3, 1.4%), lupus nephritis (2, 0.9%), C3 glomerulopathies (1, 0.5%), Henoch-Schönlein purpura nephritis (1, 0.5%), hypertensive nephropathy (1, 0.5%), multiple myeloma-associated nephropathy (1, 0.5%), idiopathic glomerular nodular sclerosis (1, 0.5%), polycystic kidney (1, 0.5%), and type of uncertain etiology (58, 26.5%). b: Renal replacement: hemodialysis (53, 24.2%), peritoneal dialysis (18, 8.2%), and kidney transplantation (12, 5.5%). c: The number of valid cases was 214, of which 159 patients survived and 55 patients died during the follow-up.

**Table 2:** The assessment of machine learning models for CKD patients with COVID-19

Model	AUC (95% CI)	Youden	Accuracy	Sensitivity	Specificity	PPV	NPV	F1	Kappa	Brier	H-L test*
LR	0.937 (0.905, 0.968)	0.775	0.886	0.891	0.884	0.721	0.960	0.797	0.719	0.090	<0.001
SVM	0.946 (0.918, 0.974)	0.781	0.881	0.909	0.872	0.704	0.966	0.794	0.712	0.082	0.968
RF	0.938 (0.908, 0.968)	0.757	0.854	0.927	0.829	0.646	0.971	0.761	0.661	0.100	0.048
XGBoost	0.925 (0.892, 0.959)	0.702	0.840	0.873	0.829	0.632	0.951	0.733	0.623	0.100	0.430

ML: machine learning; CKD: chronic kidney disease; COVID-19: coronavirus disease 2019; AUC: area under the curve; CI: confidence interval; PPV: positive predictive value; NPV: negative predictive value; H-L: Hosmer-Lemeshow; LR: least absolute shrinkage and selection operator regression; SVM: support vector machine; RF: random forest; XGBoost: extreme gradient boosting. *: P value for the Hosmer-Lemeshow test.
