# Supplementary material for: Development Of the VAMPCT Score for Predicting Mortality in CKD Patients with COVID-19
Source: Int J Med Sci. 2025 May 31;22(11):2782–91. doi: 10.7150/ijms.111558 (PMC12163619; doi:10.7150/ijms.111558)
Supplement: Supplementary file 1 — Supplementary figures and tables. [file ijmsv22p2782s1.pdf]

Table S1. Candidate predictors considered for variable selection.

Candidate predictors	Type of data	Levels of data
Demographics		
Age	Numeric	
Sex	Categorical	Male, Female.
Body mass index	Numeric	
CKD stages	Categorical	CKD 1, CKD 2, CKD 3, CKD 4, CKD 5.
Diagnosis of CKD	Categorical	IgA nephropathy, Diabetic nephropathy, Membranous nephropathy, Other chronic glomerulonephritis, Renal replacement.
Comorbidities		
Hypertension	Categorical	Yes, No.
Cardiovascular disease	Categorical	Yes, No.
Diabetes mellitus	Categorical	Yes, No.
Cerebrovascular disease	Categorical	Yes, No.
Cancer	Categorical	Yes, No.
Vaccination for COVID-19	Categorical	Unvaccinated, Partially vaccinated, Fully vaccinated.
Admission vitals		
Body temperature	Numeric	
Heart rate	Numeric	
Systolic blood pressure	Numeric	
Diastolic blood pressure	Numeric	
Finger oxygen saturation on air <90%	Categorical	Yes, No
Time from onset to admission	Numeric	
Laboratory variables	Numeric	
White blood cell	Numeric	
Neutrophil (percentage)	Numeric	
Lymphocyte (percentage)	Numeric	
Monocyte (percentage)	Numeric	
Red blood cell	Numeric	
Hemoglobin	Numeric	
Hematocrit	Numeric	
Red blood cell volume distribution width	Numeric	
Mean corpuscular volume	Numeric	
Mean corpuscular hemoglobin concentration	Numeric	
Mean corpuscular hemoglobin	Numeric	
Platelet	Numeric	
C-reactive protein	Numeric	
Interleukin-6	Numeric	
Serum potassium	Numeric	
Serum sodium	Numeric	
Serum calcium	Numeric	
Serum magnesium	Numeric	

Serum inorganic phosphorus	Numeric
Serum chloride	Numeric
Serum uric acid	Numeric
Blood glucose	Numeric
Blood urea	Numeric
Serum creatinine	Numeric
Estimated glomerular filtration rate	Numeric
Total protein	Numeric
Serum albumin	Numeric
Aspartate aminotransferase	Numeric
Alanine aminotransferase	Numeric
Gamma-glutamyltransferase	Numeric
Total bilirubin	Numeric
Direct bilirubin	Numeric
Carbon dioxide combining power	Numeric
Alkaline phosphatase	Numeric
Creatine kinase	Numeric
Creatine kinase isoenzyme	Numeric
Lactate dehydrogenase	Numeric
Prothrombin time	Numeric
Prothrombin activity	Numeric
International normalized ratio	Numeric
Thrombin time	Numeric
Activated partial thromboplastin time	Numeric
D-dimer	Numeric
Plasma fibrinogen	Numeric
Brain natriuretic peptide	Numeric
Myoglobin	Numeric
Cardiac troponin T	Numeric
Amylase	Numeric
Lipase	Numeric

CKD: chronic kidney disease; COVID-19: coronavirus disease 2019.

Table S2. The assessment of predictive scores for CKD patients with COVID-19

Model	AUC (95% CI)	Youden	Accuracy	Sensitivity	Specificity	PPV	NPV	Cutoff
VAMPCT	0.960 (0.935, 0.985)	0.799	0.904	0.891	0.909	0.766	0.961	10.5
4C Mortality	0.910 (0.871, 0.950)	0.653	0.822	0.836	0.817	0.605	0.937	10.5
HNC-LL	0.826 (0.768, 0.884)	0.587	0.790	0.800	0.787	0.557	0.921	-0.004
CURB65	0.832 (0.778, 0.886)	0.555	0.712	0.909	0.646	0.463	0.955	1.5
qSOFA	0.673 (0.602, 0.744)	0.345	0.781	0.455	0.890	0.581	0.830	0.5
MEWS	0.655 (0.578, 0.733)	0.278	0.758	0.400	0.878	0.524	0.814	2.5

CKD: chronic kidney disease; COVID-19: coronavirus disease 2019; AUC: area under the curve; CI: confidence interval; PPV: positive predictive value; NPV: negative predictive value; 4C: Coronavirus Clinical Characterisation Consortium; HNC-LL: hypertension: neutrophil count: C-reactive protein: lymphocyte count: and lactate dehydrogenase; CURB65: confusion: urea: respiratory rate: blood pressure: and age ≥ 65 years; qSOFA: quick sequential organ failure assessment; MEWS: modified early warning score.

Table S3. Risk stratification of the VAMPCT score in CKD patients with COVID-19.

Risk group	Score range	Number of patients	Mortality rate	Odds ratio (95% CI)	P value
Low risk	0 to 10	155	3.87%	81.12 (29.84, 220.57)	<0.001
High risk	≥11	64	76.56%		

CKD: chronic kidney disease; COVID-19: coronavirus disease 2019.

Table S4. The assessment of machine learning models for CKD patients with COVID-19 with different datasets

(A) Different inputed data

Model	AUC (95% CI)	Youden	Accuracy	Sensitivity	Specificity	PPV	NPV	F1	Kappa	Brier	H-L test*
LR	0.938 (0.907, 0.969)	0.769	0.881	0.891	0.878	0.71	0.960	0.790	0.709	0.089	<0.001
SVM	0.946 (0.918, 0.974)	0.781	0.881	0.909	0.872	0.704	0.966	0.794	0.712	0.082	0.968
RF	0.930 (0.896, 0.963)	0.738	0.849	0.909	0.829	0.641	0.965	0.752	0.648	0.101	0.107
XGB	0.928 (0.895, 0.961)	0.72	0.845	0.891	0.829	0.636	0.958	0.742	0.636	0.097	0.790

(B) Complete case data

Model	AUC (95% CI)	Youden	Accuracy	Sensitivity	Specificity	PPV	NPV	F1	Kappa	Brier	H-L test*
LR	0.924 (0.885, 0.964)	0.765	0.880	0.887	0.878	0.746	0.950	0.810	0.724	0.103	<0.001
SVM	0.940 (0.906, 0.974)	0.787	0.880	0.925	0.863	0.731	0.966	0.817	0.730	0.088	0.946
RF	0.921 (0.883, 0.959)	0.737	0.821	0.981	0.756	0.619	0.990	0.759	0.628	0.112	0.125
XGB	0.914 (0.875, 0.954)	0.691	0.804	0.943	0.748	0.602	0.970	0.735	0.592	0.109	0.782

ML: machine learning; CKD: chronic kidney disease; COVID-19: coronavirus disease 2019; AUC: area under the curve; CI: confidence interval; PPV: positive predictive value; NPV: negative predictive value; H-L: Hosmer-Lemeshow; LR: least absolute shrinkage and selection operator regression; SVM: support vector machine; RF: random forest; XGBoost: extreme gradient boosting.
*: P value for the Hosmer-Lemeshow test.

Supplementary Figures

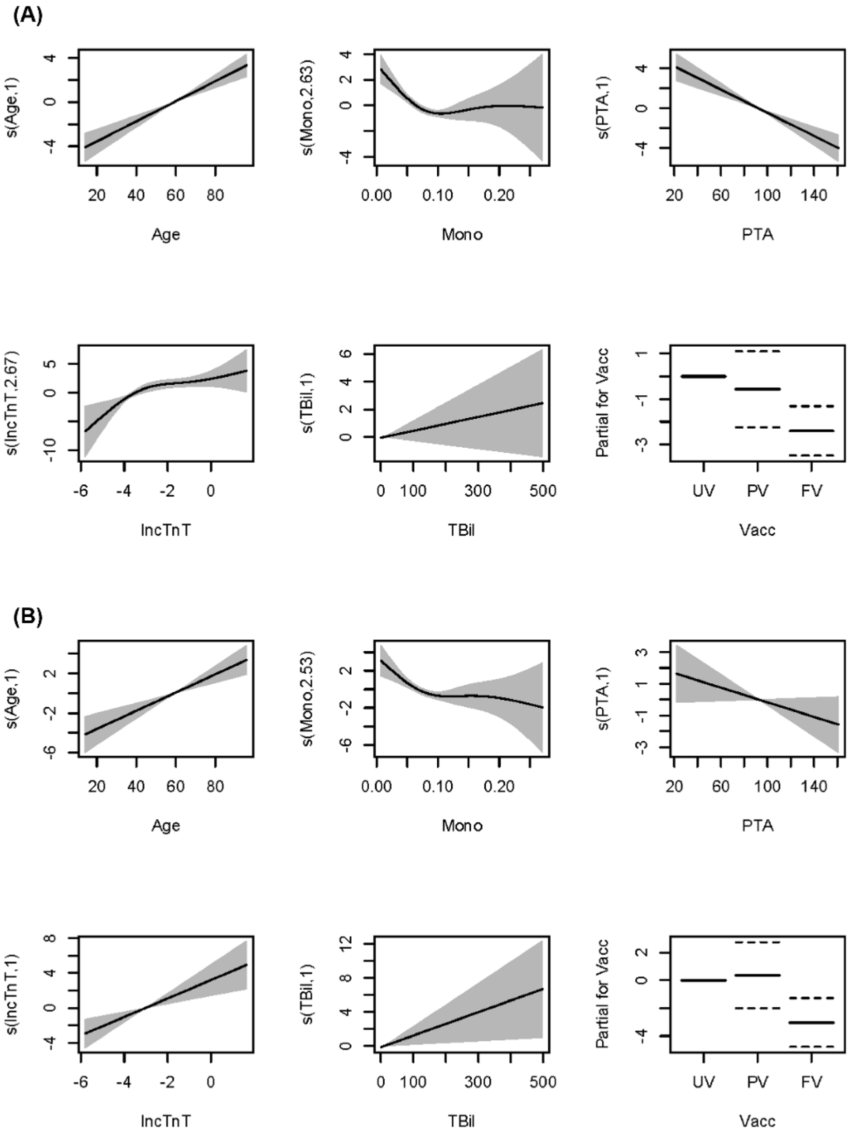

Figure S1. The generalized additive models of the selected six variables

(A) Univariable associations between different variables and the outcome

(B) Multivariable associations between different variables and the outcome

Vacc, COVID-19 vaccination status; Mono, percentage of monocyte; PTA, prothrombin activity; cTnT, cardiac troponin T; TBil, total bilirubin.

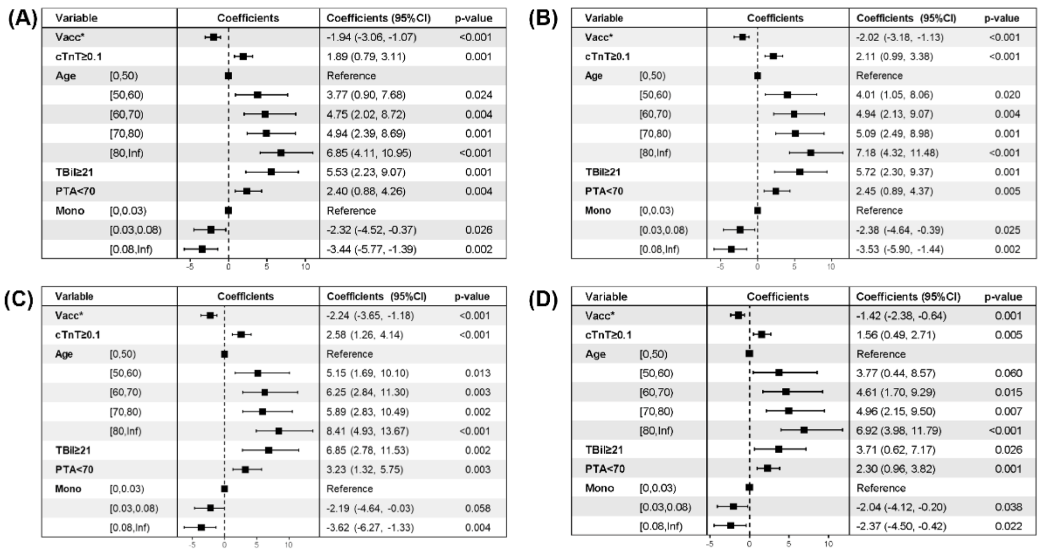

Figure S2. Sensitivity analysis: The forest plot of the VAMPCT score with six classified variables

(A) The primary scoring system.

(B) Different imputed datasets.

(C) Complete case data.

(D) In-hospital outcomes.

Vacc, COVID-19 vaccination status; Mono, percentage of monocytes; PTA, prothrombin activity; cTnT, cardiac troponin T; TBil, total bilirubin.

*The status of the COVID-19 vaccination was fitted as an ordinal variable (Unvaccinated as 0, Partially vaccinated as 1, and Fully vaccinated as 2).

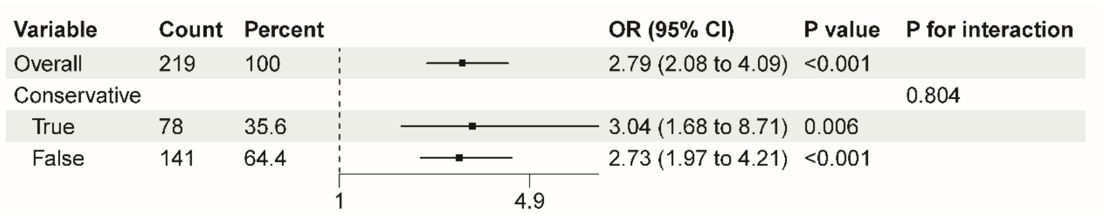

Figure S3. Subgroup analysis: The forest plot of the VAMPCT score with different treatment modality.

True: Conservative treatment-only. False: At least one non-conservative treatment.
